# Decreased prevalence of *Plasmodium falciparum* resistance markers to amodiaquine despite its wide scale use as ACT partner drug in Zanzibar

**DOI:** 10.1186/1475-2875-11-321

**Published:** 2012-09-11

**Authors:** Gabrielle Fröberg, Louise Jörnhagen, Ulrika Morris, Delér Shakely, Mwinyi I Msellem, José P Gil, Anders Björkman, Andreas Mårtensson

**Affiliations:** 1 Malaria Research Group, Department of Medicine Solna, Retzius vag 10, Karolinska Institutet, 171 77 Stockholm, Sweden; 2 Dept of Medicine, Kungälv Hospital, Kungälv, Sweden; 3 Zanzibar Malaria Control Programme (ZMCP), Ministry of Health, Zanzibar, Tanzania; 4 Drug resistance and Pharmacogenetics Group, Institute of Biotechnology and Bioengineering, Centre of Molecular and Structural Biomedicine, University of Algarve, Faro, Portugal; 5 Laboratory of Molecular Anthropology and Health, Department of Anthropology, Binghamton University, Binghamton, NY, USA; 6 Drug Resistance Unit, Section of Pharmacogenetics, Dept of Physiology and Pharmacology, Karolinska Institutet, Stockholm, Sweden; 7 Division of Global Health (IHCAR), Dept of Public Health Sciences, Karolinska Institutet, Stockholm, Sweden

**Keywords:** Malaria, *Plasmodium falciparum*, Drug resistance, Amodiaquine, Artemisinin based combination therapy

## Abstract

**Background:**

Zanzibar has recently undergone a rapid decline in *Plasmodium falciparum* transmission following combined malaria control interventions with artemisinin-based combination therapy (ACT) and integrated vector control. Artesunate-amodiaquine (ASAQ) was implemented as first-line treatment for uncomplicated *P. falciparum* malaria in Zanzibar in 2003. Resistance to amodiaquine has been associated with the single nucleotide polymorphism (SNP) alleles *pfcrt* 76T, *pfmdr1* 86Y, 184Y and 1246Y. An accumulation of these SNP alleles in the parasite population over time might threaten ASAQ efficacy.

The aim of this study was to assess whether prolonged use of ASAQ as first-line anti-malarial treatment selects for *P. falciparum* SNPs associated with resistance to the ACT partner drug amodiaquine.

**Methods:**

The individual as well as the combined SNP allele prevalence were compared in pre-treatment blood samples from patients with uncomplicated *P. falciparum* malaria enrolled in clinical trials conducted just prior to the introduction of ASAQ in 2002–2003 (n = 208) and seven years after wide scale use of ASAQ in 2010 (n = 122).

**Results:**

There was a statistically significant decrease of *pfcrt* 76T (96–63%), *pfmdr1* 86Y (75–52%), 184Y (83–72%), 1246Y (28–16%) and the most common haplotypes *pfcrt/pfmdr1* TYYD (46–26%) and TYYY (17–8%), while an increase of *pfcrt/pfmdr1* KNFD (0.4–14%) and KNYD (1–12%).

**Conclusions:**

This is the first observation of a decreased prevalence of *pfcrt* 76T, *pfmdr1* 86Y, 184Y and 1246Y in an African setting after several years of extensive ASAQ use as first-line treatment for uncomplicated malaria. This may support sustained efficacy of ASAQ on Zanzibar, although it was unexpected considering that all these SNPs have previously been associated with amodiaquine resistance. The underlying factors of these results are unclear. Genetic dilution by imported *P. falciparum* parasites from mainland Tanzania, a de-selection by artesunate *per se* and/or an associated fitness cost might represent contributing factors. More detailed studies on temporal trends of molecular markers associated with amodiaquine resistance are required to improve the understanding of this observation.

## Background

Zanzibar has recently undergone a rapid decline in *Plasmodium falciparum* transmission following combined malaria control interventions with artemisinin-based combination therapy (ACT) and integrated vector control [1,2]. In the new epidemiological context, where in vivo trials to assess ACT efficacy have been increasingly difficult to conduct due to limited number of patients, surveillance of molecular markers associated with anti-malarial drug resistance may be useful as an early warning system of development and spread of ACT resistance.

Artesunate (AS) plus amodiaquine (AQ) combination therapy (ASAQ) was implemented as first-line treatment for uncomplicated *P. falciparum* malaria free of charge to all age groups through public health care facilities in Zanzibar in September 2003. AQ and its slowly eliminated active metabolite desethylamodiaquine (DEAQ) are 4-aminoquinolines and structurally related to chloroquine (CQ). Despite the similarities and putative cross-resistance in between the compounds, AQ/DEAQ has remained more efficacious [3,4].

Resistance to CQ, AQ and DEAQ has been associated with the single nucleotide polymorphism (SNP) alleles 76T in the *P. falciparum* CQ resistance transporter (*pfcrt*) gene and 86Y in the *P. falciparum* multi drug resistance 1 (*pfmdr1*) gene [5-13]. *Pfcrt* 76T has been found within different *pfcrt* 72*–*76 haplotypes. The strongest association with AQ/DEAQ resistance has been found with *pfcrt* SVMNT, mainly found in South America and parts of Asia, while in Africa the dominating haplotype has been *pfcrt* CVIET [14,15]. Further, the SNP allele *pfmdr1* 1246Y and the haplotype *pfmdr1* (a.a. 86, 184, 1246) YYY have been selected for among recurrent infections after treatment with AQ monotherapy and ASAQ combination therapy in East Africa [10,16]. Selection and accumulation of these SNPs in the parasite population over time could potentially threaten ASAQ efficacy.

The aim of this study was to assess whether prolonged use of ASAQ as first-line anti-malarial treatment selects for *P. falciparum* SNPs associated with resistance to the ACT partner drug AQ.

## Methods

The prevalence of *pfcrt* 76T, *pfmdr1* 86Y, 184Y and 1246Y were compared in pre-treatment blood samples collected on filter papers (3MM^®^, Whatman, UK). Samples were collected from individuals with uncomplicated *P. falciparum* malaria, residing in North A (Unguja Island) and Micheweni (Pemba Island) districts in Zanzibar. Patients were enrolled in clinical trials conducted just prior to the introduction of ASAQ in 2002–2003 (n = 208) [16,17] and seven years after wide scale use of ASAQ in 2010 (n = 122) (Shakely *et al.* 2012, *unpublished data*). Malaria diagnosis was confirmed by blood smear microscopy and rapid malaria diagnostic (RDT), respectively.

DNA extraction and genotyping of samples from 2002–2003 and 2010 was performed with similar methods which have been described elsewhere [16,17]. In summary, DNA was extracted by ABI PRISM 6100 Nucleic Acid PrepStation^TM^ (Applied Biosystems, USA) and genotyping analysis of *pfcrt* K76T, *pfmdr1* N86Y, Y184F and D1246Y were performed through previously described PCR-RFLP methods [5,16,18]. All PCR reactions contained 1 × *Taq* polymerase reaction buffer, 2.5–3 mM magnesium chloride, 0.2 mM dNTP, 0.5–1 μM of each primer and 1.25 units of *Taq* DNA polymerase (Promega Corporation, USA). RFLP reaction contained 1 × NEBuffer 1/3, 0–1 × BSA and 10 U/reaction of ApoI, Tsp509 I or EcoR V restriction enzymes. PCR-RFLP products were visualized under UV transillumination (GelDoc 2000, BioRad, Hercules^®^, CA, USA) after 2–2.5% agarose gel electrophoresis and ethidium-bromide staining.

A mixed infection was considered to contain two *P. falciparum* strains, contributing with one of each SNP alleles during PCR-RFLP. In the haplotype analyses all isolates including mixed SNP results at more than one position were excluded. Allele and haplotype prevalences between 2002–2003 and 2010 were compared by chi square tests (SigmaPlot^®^ 11.0, Systat Software Inc, USA). Statistical significance was defined as p < 0.05.

The clinical trials were performed in accordance with the Declaration of Helsinki [19] and Good Clinical Practice [20]. Informed written consent was obtained from the parents/guardians of all enrolled participants. Ethical approvals were obtained from the relevant ethical committees in Zanzibar at the time of the trials (ZHRC/GC/2002, ZMRC/RA/2005 and ZAMEC/ST/0021/09) and the Medical Ethics Committee at Karolinska Institutet (KI Dnr 03–753, KI Dnr 2005/57-31) and the Regional Ethics Committee in Stockholm, Sweden (2009/387-31).

## Results

DNA was successfully extracted from 117/122 (96%) of the blood samples from 2010.

**Figure 1:**
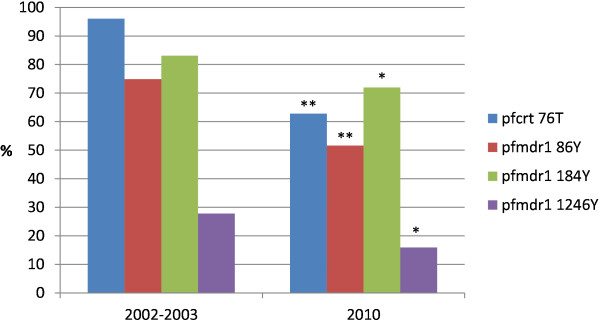
**SNP frequencies in Zanzibar before (2002–2003) and seven years after (2010) ASAQ implementation.** Asterisk (*) and (**) indicate statistically significant differences of p < 0.05 and p < 0.001, respectively.

The individual SNP prevalences before (2002–2003) and seven years after (2010) ASAQ implementation in Zanzibar are shown in Figure 1. There was a statistically significant decrease in the prevalence of *pfcrt* 76T from 195/203 (96%) to 76/121 (63%) (p < 0.001), *pfmdr1* 86Y from 170/227 (75%) to 64/124 (52%) (p < 0.001), 184Y from 197/237 (83%) to 89/123 (72%) (p = 0.024) and 1246Y from 72/259 (28%) to 18/113 (16%) (p = 0.020).

**Figure 2:**
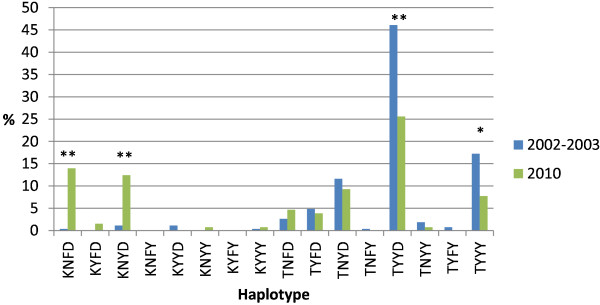
**Haplotype (*pfcrt*K76T,*pfmdr1*N86Y, Y184F and D1246Y) frequencies in Zanzibar before (2002–2003) and seven years after (2010) ASAQ implementation.** Asterisk (*) and (**) indicate statistically significant differences of p < 0.05 and p < 0.001, respectively.

The haplotype (*pfcrt* K76T/*pfmdr1* N86Y, Y184F, D1246Y) prevalence before and seven years after ASAQ implementation are shown in Figure 2. The most common haplotypes before implementation were TYYD and TYYY. Their respective prevalence decreased from 123/267 (46%) to 33/129 (26%) (p < 0.001) and 46/267 (17%) to 10/129 (8%) (p = 0.017). On the other hand, KNFD and KNYD increased over the time period from 1/267 (0.4%) to 18/129 (14%) (p < 0.001) and 3/267 (1%) to 16/129 (12%) (p < 0.001).

## Discussion

This is the first observation of a decreased prevalence of *pfcrt* 76T, *pfmdr1* 86Y, 184Y and 1246Y in an African setting after several years of extensive ASAQ use as first-line treatment for uncomplicated malaria. This may support sustained efficacy of ASAQ on Zanzibar, although it was unexpected considering that all these SNPs have previously been associated with AQ/DEAQ resistance.

The underlying factors of these results are unclear. Genetic dilution by imported *P. falciparum* parasites from for example mainland Tanzania could represent a contributing factor. Even though Zanzibar is a part of Tanzania, they are independent in some issues e.g. the malaria control programme. Mainland Tanzania implemented artemether-lumefantrine (Coartem^®^) as first-line treatment in 2006 when this ACT was widely manufactured, price had reduced and studies were shown it was safe to give children below ten kg. Artemether-lumefantrine, has shown to select for the opposite alleles i.e. *pfcrt* 76K, *pfmdr1* 86N, 184F and 1246D [21-24].

Another contributing factor may be that AS *per se* potentially selects for *pfcrt* 76K, *pfmdr1* 86N and 1246D, which have been associated with decreased susceptibility to the artemisinins in vitro [25,26]. Importantly however, no such selection has been shown after monotherapy with artemisinin derivatives in vivo.

A third contributing factor may be that SNPs associated with AQ resistance cause a fitness cost to the parasite, which would affect the selection pattern under different drug pressures. In competition experiments between modified isogenic clones, only differing in the *pfmdr1* 1246 position, *pfmdr1* 1246Y was found to be associated with a substantial fitness cost to the parasite (Fröberg *et al.* 2012, *unpublished data*). This could also apply on the other SNPs and also explain the haplotype results in this study. Before ASAQ implementation the most common haplotype was TYYD, indicating that the previous first-line treatment i.e. CQ mainly selected for *pfcrt* 76T, *pfmdr1* 86Y and 184Y. The second most common haplotype was TYYY, where *pfmdr1* 1246Y has mainly been associated with AQ/DEAQ resistance. Seven years later a significant selection of KNFD and KNYD was observed. Hence, the individual SNPs *pfcrt* 76T, *pfmdr1* 86Y and 1246Y rarely exist alone, suggesting that they may be associated with a significant fitness cost and support each other in a possibly synergistic and/or compensatory relationship, whereas *pfmdr1* 184Y do exist alone and might not largely affect fitness.

Finally, even though these SNPs have been selected for after AQ/ASAQ treatment, the association with AQ/DEAQ resistance may not be that strong that it will spread with prolonged wide-scale use of ASAQ.

## Conclusions

Seven years after wide scale use of ASAQ as first-line treatment in Zanzibar, SNPs associated with AQ/DEAQ resistance have not been selected for. Instead, the prevalence of these SNPs has decreased, which may support sustained efficacy of this ACT as first-line treatment in Zanzibar. However, the results were unexpected, which calls for more detailed studies of temporal trends of molecular markers associated with AQ/DEAQ resistance both among symptomatic and asymptomatic *P. falciparum* infections to improve the understanding of this observation.

## Competing interests

The authors declare that they have no competing interests.

## Authors’ contributions

GF, JPG, AM and AB conceived and designed the study. DS, AM and MIM carried out the field work. GF, LJ and UM carried out the molecular analyses. GF, LJ, UM, JPG, AB and AM analyzed the data. GF and AM wrote the manuscript. All authors revised and approved the final manuscript.
